# Pathology and Therapeutics of Dead Teeth

**Published:** 1897-02

**Authors:** G. H. Claude


ARTICLE III.

### PATHOLOGY AND THERAPEUTICS OF DEAD TEETH.

BY DR. G. H. CLAUDE.

**Read at Union Meeting of Maryland State Dental Association and Washington City Dental Society.**

The author defines a tooth as dead when the pulp has been destroyed. Respecting the morbid anatomy of such cases, he said: When a pulp dies, the pericementum—that portion of the periosteum at the root apex, reflected upon the root from that lining the alveolus—becomes congested and, unless early interference is had, suppuration supervenes. First, the pericementum becomes distended and partially detached from the root, pus generates in the sac thus formed and becomes a foreign element, which must be thrown off, and, if influenced by gravity only, would discharge by the shortest route; but it is directed therefrom by the anatomical structure of the environments. Pus once formed and the periosteal sac containing it broken, it must then find its way through the bony structure until it reaches the periosteum covering the outside of the maxila when it becomes again arrested by this tough, elastic membrane, causing much distension and inflammation of the surrounding tissues. The pressure of the constantly increasing generation of gas, from decomposition of the pulp and pus-secretion from the sac thus formed, causes a thinning of the soft structures until an opening is established, when the trouble subsides in a few days, leaving a permanent fistula unless interfered with.

Whatever the treatment—extraction, which is seldom necessary, or cure by other means—morbid changes have already taken place which will remain through life. The periosteum becomes thickened, the cementum nodular, if inflammation has preceded the death of the pulp, and the bony structure has become somewhat thickened.

The color of a tooth is not altered by the death of the pulp if the debris is all removed and it is properly filled before the discoloration takes place. It remains a useful and ornamental part of the human organism, and if anything it is more adherent and firmly fixed in its socket than if the pulp were alive.

The most prominent symptom of the partial death or approaching death of the pulp is a not well-localized, intense pain on the affected side of the jaw, described often as neuralgia, the pain intermittent in character, sometimes described as thumping or hammering, increased in intensity when in the reclining position.

It is often difficult to determine in the early stages the tooth thus affected, and most frequently the pain is described by the patient to be in some other tooth than the one causing the trouble. I was called to see a patient a short time since who had a dentist extract the upper second bicuspid, which was perfectly sound, only to get a few moments' relief, probably from loss of blood. Shortly afterward the attack returned with renewed violence. He sent for his physician and was laid up in bed, resting only moments at a time while under the influence of anodynes. After being under anodyne and tonic treatment for three days he sent for me. Upon examination I could see no fillings or cavities in his teeth. The upper right lateral incisor appeared a little dull in color, and had a slight fracture of enamel near the cutting-edge. A year previous to this I had examined his teeth, and he reminded me that I had told him then it looked like a dead tooth. On feeling in the roof of his mouth just above the root of this tooth, I found it exquisitely sensitive to pressure, and detected some distension, softening, and fluctuation. I brought my dental engine to his house and drilled through the palatal surface into the pulp canal, and pus flowed freely through the openings. His relief was immediate. After two weeks when the discharge had ceased, I filled the root, with no subsequent trouble. The pulp was devitalized by a blow when he was a child, and judging from the size of the apical foramen, before the tooth was thoroughly developed.

Cases of this sort are of too frequent occurrence where good teeth are extracted on the demand of the patient, without the dentist using proper discrimination.

When in doubt, a delay of a few days or hours may establish beyond peradventure the diagnosis, and teeth may be saved which otherwise are ruthlessly extracted. Patients will nearly always consent to put up with the suffering for a time if alleviated by remedies. A careful examination will always raise a suspicion of the offending tooth, and often settle the diagnosis from the pain on percussion, looseness, insensibility at cervical margin, and response sometimes to heat and cold. A partially dead pulp will respond to heat and cold even more actively than a tooth in a normal condition, There may be no pain on cold being applied, but the pain will be intensified by the application of heat. This is a very important point in a discriminating diagnosis.

The pulp once dead, the cavity should be made large enough to afford access to all of the roots, and they should be cleaned out thoroughly to the apex, disinfected and filled.

His method of filling the pulp canal is that described by Dr. N. S. Shields—filling with gold or other substance that will not absorb moisture.—*Dental Cosmos.*

